# Validation of the Finite Element Model versus Biomechanical Assessments of Dental Implants and Total Knee Replacements

**DOI:** 10.3390/bioengineering10121365

**Published:** 2023-11-27

**Authors:** Kwan-Su Kang, Kwang-Min Park, Jin-Woo Ahn, Min-Young Jo, Yu-Rim Oh, Jin-Ho Youn, Jeong-Woo Lee, Dong-Young Je, Tae-Gon Jung

**Affiliations:** 1 Medical Device Development Center, Osong Medical Innovation Foundation, Cheongju-si 28160, Republic of Korea; oioi1578@kbiohealth.kr (K.-S.K.); kmpark@kbiohealth.kr (K.-M.P.); jwahn@kbiohealth.kr (J.-W.A.); 2 Department of Biomedical Engineering, School of Medicine, Chungbuk National University, Cheongju-si 28644, Republic of Korea; 3 Department of Biomedical Engineering, College of Biomedical Science & Engineering, Inje University, Gimhae-si 50834, Republic of Korea; dud5090@gmail.com (M.-Y.J.); oyl101526@gmail.com (Y.-R.O.); yjinho0305@gmail.com (J.-H.Y.); affectionalhome@gmail.com (J.-W.L.); helleh96@gmail.com (D.-Y.J.)

**Keywords:** total knee replacement, dental implant, finite element method

## Abstract

Computer modeling and simulation (CM&S) technology is widely used in the medical device industry due to its advantages such as reducing testing time and costs. However, the developer’s parameter settings during the modeling and simulation process can have a significant impact on the results. This study developed a test model for the rotational shear strength of dental implants and the constraint force of total knee replacements based on CM&S technology and proposes ideal parameters to ensure reliability. For dental implants, the load area and sliding contact conditions were considered, and for total knee replacements, the friction coefficient, medial–lateral displacement, valgus–varus rotation, and elastic modulus were considered. By comparing the simulation results and mechanical tests, boundary conditions with an error rate of less than 1.5% were selected. When a jig (gripper and collector) was applied with the same boundary conditions, an error rate of 48~22% occurred; otherwise, it was confirmed that the error rate was within 10~0.2%. The FE model was verified with an error of 2.49 to 3% compared to the mechanical test. The friction coefficient variable had the greatest influence on the results, accounting for 10 to 13%, and it was confirmed that valgus–varus rotation had a greater influence on the results than medial–lateral displacement. Relatively, the elastic modulus of the insert had the least effect on the results. These research results are expected to make CM&S techniques useful as a medical device digital development tool (M3DT) in the development of total knee replacements and dental implants.

## 1. Introduction

In the medical device industry, computational modeling and simulation (CM&S) techniques are the most promising solution to replace animal testing by securing in silico models and databases. The use of CM&S in designing and developing medical devices is gradually expanding in Korea and other advanced countries such as the US (FDA). Finite element analysis is a representative computer simulation technique and has been used to evaluate the mechanical performance of medical devices and as a supplement to bench testing medical device products [1,2].

Currently, based on ASME V&V 40-2018, Assessing the Credibility of Computational Modeling Through Verification and Validation: Application to Medical Devices, the American Society of Mechanical Engineers (ASME) provides a framework for establishing the credibility requirements of MDDTs, in which the error rate of finite element analysis and experimental data is within 5% [3,4,5].

Also, international standards that apply CM&S and are recognized as medical device development tools (MDDTs) include ASTM F2996-13 (Standard Practice for Finite Element Analysis (FEA) of Non-Modular Metallic Orthopaedic Hip Femoral Stems) and ASTM F3161-16 (Standard Test Method for Finite Element Analysis (FEA) of Metallic Orthopaedic Total Knee Femoral Components under Closing Conditions), in which finite element analysis is used to determine the stresses and strains that occur in medical devices when loads are applied and assess worst cases [6,7].

Although CM&S technology is actively applied in the development of medical devices in artificial knee joints and artificial hip joints, the application of CM&S technology in dental implants is relatively low. The finite element analysis of dental implants is mainly used to analyze biomechanical characteristics [8,9,10]. Finite element analysis can be used in dental implant development because it can construct modeling in the same way as standard testing. If the reliability of the finite element model is verified, it can replace performance evaluation through bench testing, thereby reducing time and economic losses [11].

The first test (Part 1) to which CM&S was applied in this study was the rotational strength test of dental implants. This test is standardized in ISO/TS 13498 [12] to evaluate the stability against rotational force generated by implant insertion or mastication movement between the fixture and the abutment. Along with ISO 14801 (Dynamic loading test for endosseous dental implants) [13], it is a representative test that evaluates the stability of dental implants. In the development of dental implants, various mechanisms are considered between fixtures and abutments. However, conducting stability evaluations through bench testing requires considerable time and economic cost to evaluate stability. Therefore, it is necessary to apply finite element analysis using CM&S technology.

The second test (Part 2) is TKR’s constraint test. This test evaluates the stability of artificial knee joints by evaluating their resistance to dislocation and is specified as a standard in ASTM F1223-20 [14]. In addition, ASTM F2083-21 [15] (Standard Specification for Knee Replacement Prosthesis) specifies that evaluation can be performed using computer modeling validated through mechanical testing. This standard was selected along with the rotational shear strength test of dental implants.

The target medical device test for this study was selected as a constraint test for total knee replacement and a rotational strength test for dental implants. The constraint test for total knee replacement is specified as a standard specification in ASTM F2083-21 [15] as a test to evaluate the safety of joint dislocation. However, in these tests, the axial compressive load and translational displacement are control and adjusting variables that are not clearly stated in the standard. Consequently, repeated tests must be performed until accurate results are obtained, as different results may appear depending on the values of these variables [14,16]. The rotational strength test of a dental implant must verify its stability against the rotational force generated by the masticatory movement between the fixture and the abutment.

In this study, we developed a finite element model for the rotational strength testing of dental implants and constraint testing of TKR using CM&S technology. The model was then verified through mechanical testing and a comparison of the obtained results. The impact of the parameters derived in this process on the simulation results was evaluated. Lastly, this study sought to confirm the applicability of CM&S technology as a medical device digital development tool (M3DT) to evaluate the development, safety, and effectiveness of dental implants and artificial knee joint medical devices.

## 2. Materials and Methods

### 2.1. Part 1: Dental Implant

#### 2.1.1. FE Model of Dental Implants

**Figure 1:**
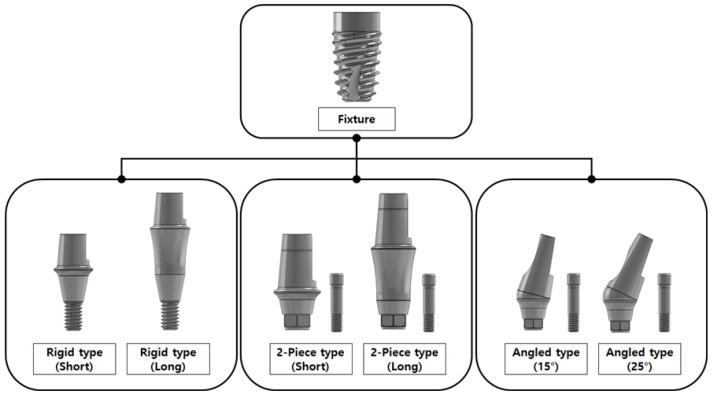
Dental implant specimens used in the experiment.

The dental implants used in the experiment were produced by a manufacturer in Korea. As shown in Figure 1, the implants were divided into the fixture (submerged type) and abutment (rigid, 2-piece, angled type), and six types of products were prepared according to two abutment sizes (long and short length/high (25°) and low (15°) angle) for worst-case assessment.

**Figure 2:**
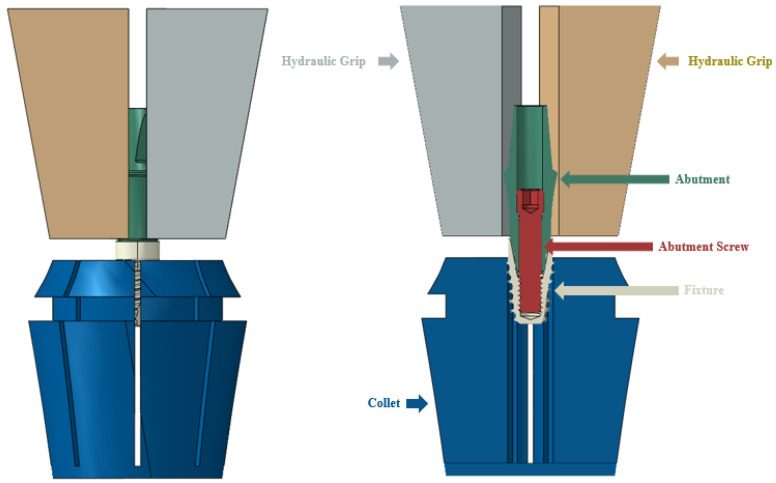
FE model of dental implant and jig (2-piece long-type model).

Six dental implant finite element models were created (identical to the specimens used in the mechanical test). Similar to the mechanical test environment, jig collet and flat grip models were also implemented for fixing the test specimens. The fixture was made of titanium grade 4, the abutment and abutment screw had non-linear properties of Ti-6Al-4V, and the jig had stainless linear properties, as shown in Table 1. The 3D model was created using SolidWorks (SolidWorks Corp., Concord, MA, USA). The finite element analysis model was implemented using Abaqus (ABAQUS, Dassault System, vélizy-villacoublay, France), as shown in Figure 2 and Table 2.

#### 2.1.2. Simulation

**Figure 3:**
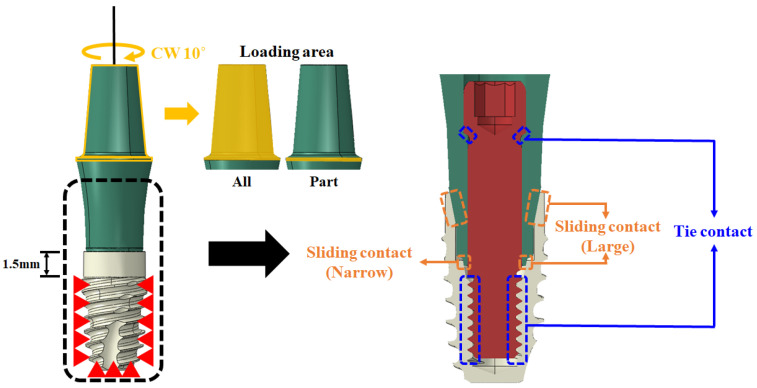
Contact condition of dental implant.

The degree of freedom of the entire threaded portion 1.5 mm below the fixture was constrained in all directions. This setup replicates the mechanical test, based on the 2-piece-type (long) model, which is the most used in dental implant procedures. These conditions were derived to establish the ideal finite element analysis conditions for testing the torsional strength of dental implants. Two variables were set for the load. These were labeled “All” and “Part”. All pertained to the largest diameter part of the abutment (from the largest diameter portion to the top), and the term “Part” specifically referred to the largest diameter portion. A 10° rotational displacement was then applied clockwise. In addition, a tie contact condition was applied to the threaded portion of the abutment and fixture. This was under the assumption that they were completely connected, and a sliding contact condition with a 0.2 friction coefficient was applied to some parts, as shown in Figure 3 and Table 3, to configure six variables [21].

**Figure 4:**
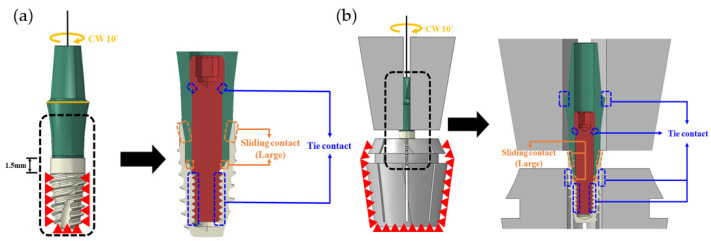
Finite element analysis environment with or without jig: (**a**) jig not applied; (**b**) jig applied.

Based on the 2-piece-type (long) model, the ideal contact and loading conditions were derived by comparing the finite element analysis and mechanical test results according to the six variables. These were then applied to all dental implant models. In addition, a model was implemented, as shown in Figure 4, for a comparative analysis both with and without jigs, and a tie contact condition was applied, assuming a complete coupling between the jig and the implant.

#### 2.1.3. Mechanical Test Method for FE Model Validation

**Figure 5:**
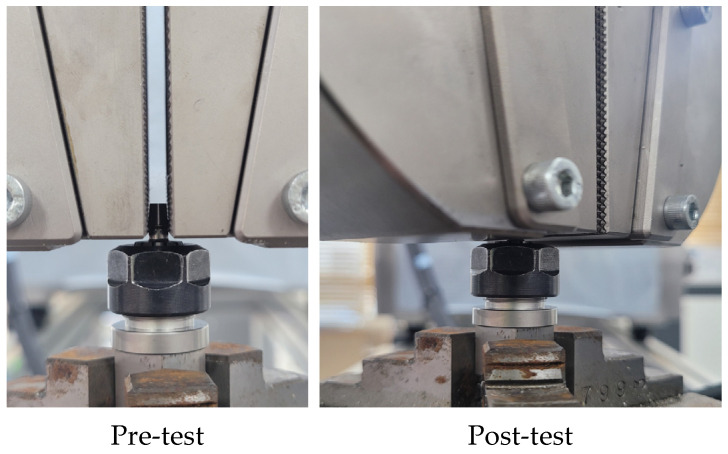
Images of the fixed test specimen.

The mechanical test was performed based on ISO/TS 13498 [12] using a universal testing system (Bionix 858, MTS Systems Corp., Eden Prairie, MN, USA) to evaluate the torsional strength of dental implants [12]. The entire threaded portion 1.5 mm below the fixture was fixed using a collet, as shown in Figure 5, and the largest diameter portion of the abutment was fixed with a flat grip. The test speed was set to 10°/min, rotating clockwise up to 10°, and the torque–displacement data were acquired at a 50 Hz frequency. The yield angle (°) and yield torque (Nm) were derived using the 2° offset method from the obtained torque–displacement graph. The reaction torque–angle curve was calculated through finite element analysis and compared with the yield torque corresponding to the yield angle in the mechanical test.

### 2.2. Part 2: Total Knee Replacement (TKR)

#### 2.2.1. FE Model of TKR

**Figure 6:**
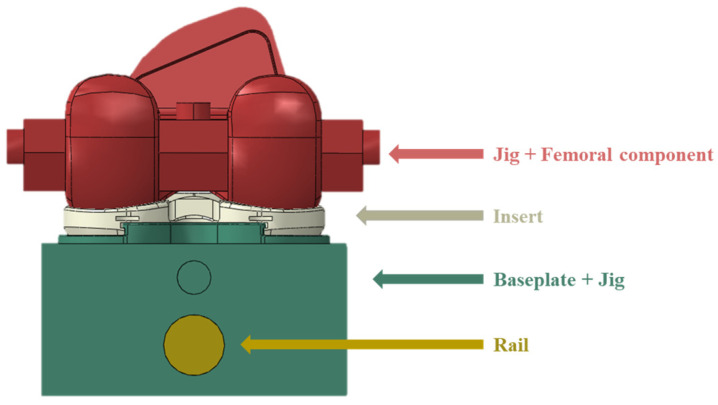
Modeling of total knee replacement and jig rail.

The total knee replacement used in this study is the DePuy Attune (DePuy Synthes, Warsaw, IN, USA), which is commercially available and widely used in clinical practice. The three components of the total knee replacement (femoral component, insert, and baseplate) were 3D modeled with a 3D scanner (MetraSCAN BLACK Elite, Creaform, CA, USA). The femoral component jig, baseplate jig, and rail were designed using SOLIDWORKS 2016 (Dassault System, vélizy-villacoublay, France) to assign loads and boundary conditions to the joint. Simplification was performed by integrating the femoral component with the femoral component jig and the baseplate with the baseplate jig to reduce finite element analysis time and errors (Figure 6). The material properties of each component of the finite element model are shown in Table 4 [22,23].

#### 2.2.2. Simulation

Various boundary conditions were applied considering the structural characteristics of the artificial knee joint and its movement. Scenario 1 (Case 1) considered the friction coefficient between the femoral component and the insert. Scenario 2 (Cases 2–5) considered the difference according to the *X*-axis (medio-lateral) and *Y*-axis (valgus–varus) degrees of freedom, and Scenario 3 (Case 6) considered the elastic modulus of the UHMWPE material used for the insert as a parameter.

**Figure 7:**
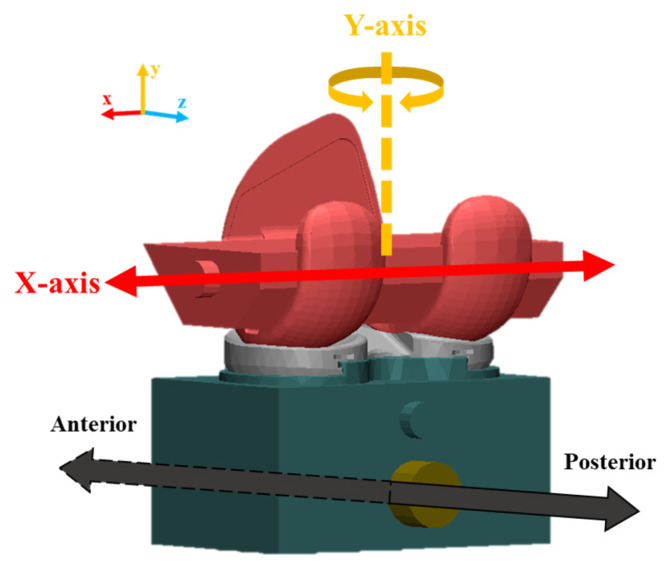
DOF of anterior–posterior draw test.

In Case 1, the coefficient of friction was varied by 0.05, ranging from 0 to 0.3, to determine the coefficient of friction between the femoral component and the insert. The *X*-axis (medio-lateral) and *Y*-axis (valgus–varus) were fixed. In Cases 2 to 5, the degrees of freedom for the *X*-axis (medio-lateral) displacement and the *Y*-axis (valgus–varus) rotation were applied as parameters (Figure 7). In Case 5, the elastic modulus of the insert was applied at 855 MPa, 900 MPa, and 945 MPa, respectively (Table 5).

**Figure 8:**
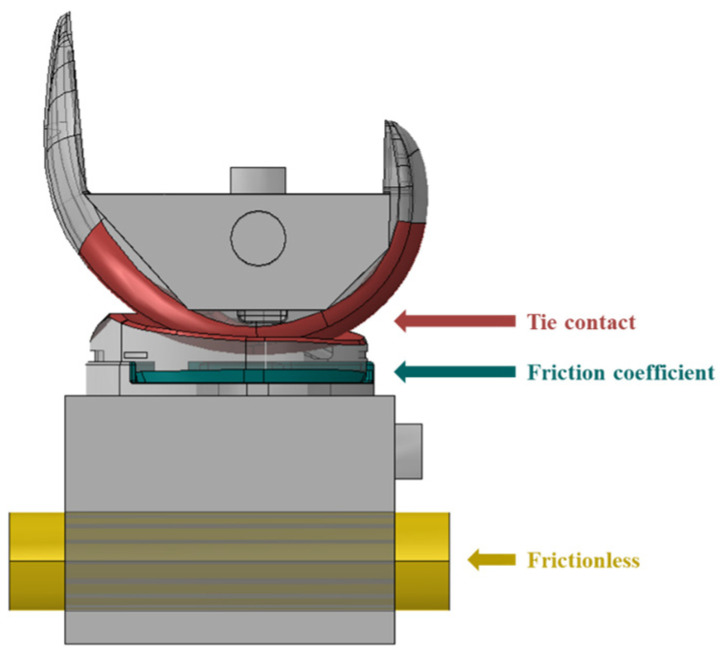
Contact condition of FE model.

A tie contact condition was applied as a common boundary condition, assuming that the insert and baseplate were connected and there was no friction between the baseplate jig and rail (Figure 8). As for the loading conditions, a vertical load of 710 N was applied to the upper jig based on ASTM F1223-20 [14]. The lower jig was then moved backward in the *X*-axis direction, by ten steps of 1 mm each, totaling 10 mm. All simulations were run using ABAQUS (Dassault System, vélizy-villacoublay, France), a commercial finite element-based software package. The elements of each component were implemented as C3D10 tetrahedral elements, and the analysis was performed by applying isotropic and homogeneous material properties.

#### 2.2.3. Mechanical Test for FE Model Validation

**Figure 9:**
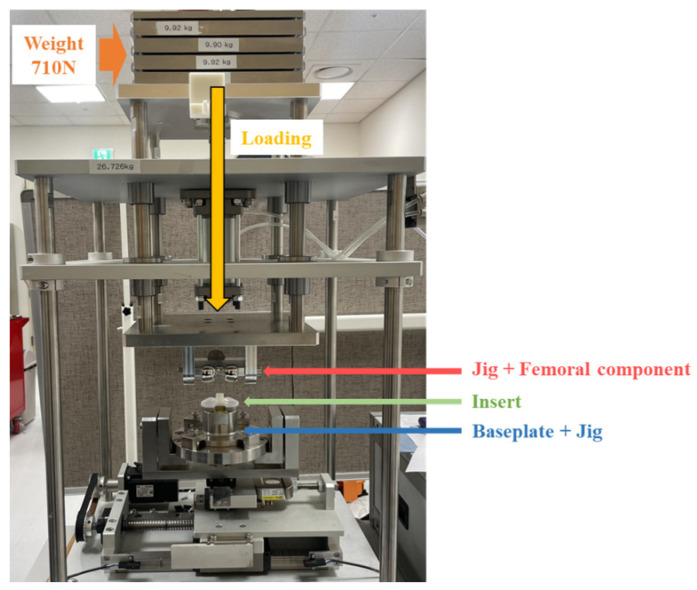
Total knee replacement mechanical test system.

The finite element model was validated through mechanical tests using a proprietary total knee replacement mechanical testing system (Figure 9). The system was designed to apply a constant vertical load using a weight at the top and implement medio-lateral movement and valgus–varus rotation at the bottom. The displacement and reaction force were obtained by moving the lower plate with a motor to implement the dislocation of the artificial knee joint. The baseplate was fixed to a dedicated port using resin (Vertex Trayplast NF; Vertex Dental BV, NL), and the port was mounted on the testing system. Anterior and posterior constraint tests were conducted at 0° flexion of the femoral component, with the *X*-axis (medio-lateral) and *Y*-axis (valgus–varus) fixed as in the simulation. As for vertical loading, a weight equivalent to an adult’s body weight (70 kg) was used based on ASTM F1223-20 [14]. The dislocation of the knee joint was realized by moving the lower plate at a speed of 10 mm/sec to dislodge the insert. The displacement and force generated were recorded at a frequency of 10 times per second and was then compared with the simulation results.

## 3. Results

### 3.1. Part 1: Dental Implant

#### 3.1.1. Mechanical Test Results

**Figure 10:**
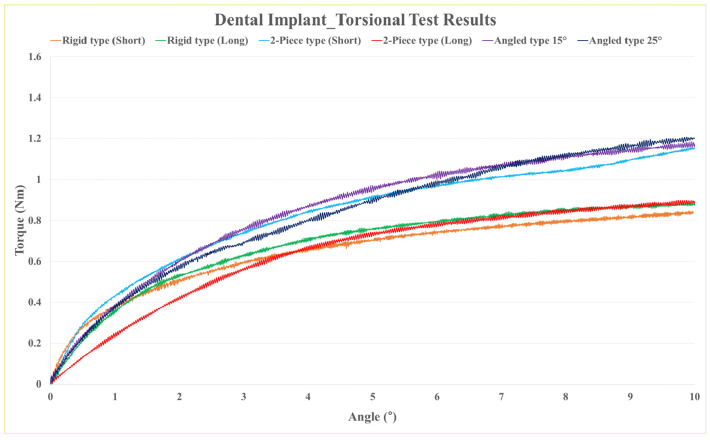
Graph of mechanical test results on dental implant torsional strength.

Table 6 shows the mean and standard deviation of the dental implant torsion test results, and Figure 10 shows the results in a graph. The rigid-type (short) model showed the lowest torsional yield strength (0.61 ± 0.05 Nm), and the angled-type (15°) model showed the highest (0.97 ± 0.02 Nm).

#### 3.1.2. Dental Implant Simulation

**Figure 11:**
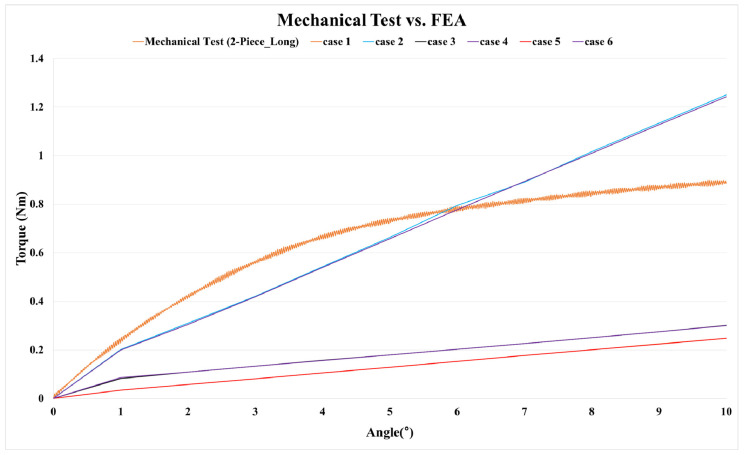
Graph of mechanical test and FEA (loading and sliding contact condition; case 1–6) results.

According to the mechanical test results of the two-piece-type (long) model, the average yield angle was 6.02° and the average yield strength was 0.77 Nm. When performing the finite element analysis according to the six variables, Table 7 shows the reaction force (Nm) at 6° and the error rate with the two-piece-type (long) model. Figure 11 shows the results in a graph. In cases of the loading and sliding contact condition variables, Cases 2 and 4, the error rate was within 5%, and Case 4 showed the lowest error rate (1.3%). In the mesh convergence study, the model of Case 4, which showed the smallest error rate, was conducted by refining the element size until the reaction force variables converged to less than a 5% change from one mesh size to the next. The element sizes were 0.26, 0.24, 0.22, 0.2, 0.18, 0.16, and 0.14, and converged from 0.24 mm to 0.16 mm with an error rate of 5%.

The loading and sliding contact conditions of Case 4 were applied to all models. Table 8 shows the finite element analysis results with and without a jig for each model. Without a jig, the error rate for the yield strength was within 5% in all models. However, when the jig was employed, the error rate ranged from 21.65% to 48.05%.

**Figure 12:**
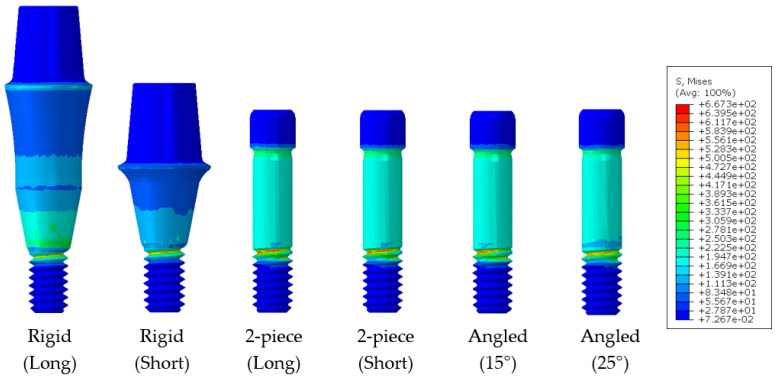
Stress distribution of abutment screw and abutment.

The stress distribution that occurs in the abutment screw (a vulnerable part of the dental implant structure) under rotational displacement was investigated. As a result, the peak von Mises stress (PVMS) occurred at the beginning of the screw thread in all models, as shown in Figure 12.

### 3.2. Part 2: Total Knee Replacement

#### 3.2.1. Finite Element Modeling

Finite element models of the artificial knee joint components (femoral component, insert, baseplate) and jig (femoral component jig, baseplate jig, rail) were created using the ABAQUS software (Table 9). The element size of the contact surface between the femoral component and the insert was set in detail.

#### 3.2.2. Validation of the Finite Element Model

**Figure 13:**
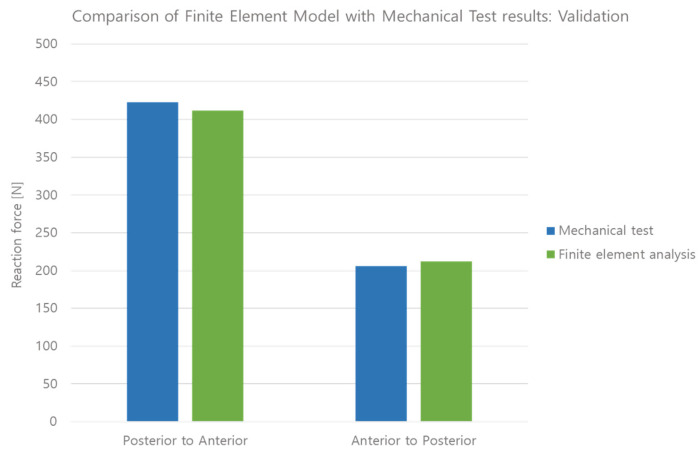
Comparison of simulation with mechanical test results: validation.

In the mechanical test, the force required to dislodge the knee joint from the posterior to the anterior was 422.57 N, and the force to dislodge the knee joint from the anterior to the posterior was 205.87 N. Based on a 0.05 coefficient of friction [24], the simulation results showed that the force required to dislodge the knee joint from the posterior to the anterior was 412.01 N, and the force required from the anterior to the posterior was 212.06 N (Figure 13). The errors of the mechanical test and simulation results were 2.49% and 3.00%, respectively, both within the 5% validation error rate suggested by ASME V&V 40 [5].

#### 3.2.3. TKR Constraint Simulation

**Figure 14:**
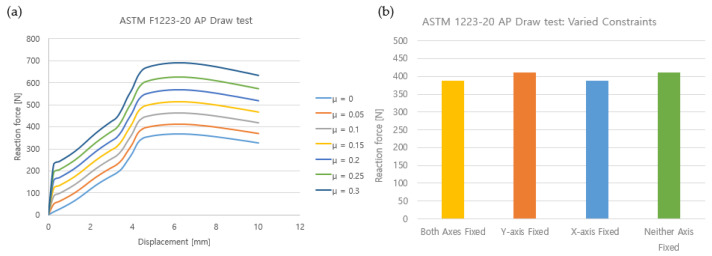
Simulation results: (**a**) friction coefficient variation; (**b**) *X*-axis, *Y*-axis constraints.

The results of the constraint test according to the friction coefficient between the femoral component and the insert showed that the higher the friction coefficient, the higher the force required to dislodge the TKR. As the coefficient of friction increased by 0.05 from 0 to 0.3, the maximum constraint increased by about 10~13% in each step, from 349.34 N to 679.65 N (Figure 14). Figure 14 shows the difference in results for rotation in the *Y*-axis (medial–lateral) direction and the *X*-axis (valgus–varus) depending on the degree of freedom constraints. The maximum constraint was 388.95 N when constraining both the Y- and X-axes. The maximum constraint was 410.1 N when only the *Y*-axis was constrained, and 388.21 N when only the *X*-axis was constrained. Lastly, when neither the *Y*-axis nor the *X*-axis were constrained, the maximum constraint was 412.01 N. In terms of the difference in the elastic modulus of the insert, the lower the elastic modulus, the higher the maximum constraint. When the elastic modulus was 855 MPa, 900 MPa, and 945 MPa, the maximum constraint was 514.19 N, 514.62 N, and 515 N, respectively, showing a difference of up to 0.08%.

## 4. Discussion

According to the Ministry of Food and Drug Safety (MFDS), dental implants and TKR are classified as Class 3 implantable medical devices. Therefore, appropriate performance tests and adherence to performance levels are necessary according to domestic and international test standards. However, significant time and economic losses occur in the repeated process of specimen production, performance testing, modification, and verification to achieve desired performance levels [24]. In recent years, CM&S has been used in many fields as a digital development tool to replace bench testing and evaluate medical devices. However, as there is a lack of research on M3DTs based on validated CM&S techniques for dental implants, this study aimed to present a validated digital development tool for this field.

### 4.1. Part 1: Dental Implants

As an implantable medical device, dental implants must be biocompatible and have sufficient mechanical and engineering strength to withstand masticatory loads to work properly. Appropriately, many previous studies have emphasized the mechanics of dental implants as a factor in their success [25,26,27]. Rieger et al. emphasized that implants should be designed to distribute stresses. Holmgren et al. reported that the wider the maximum diameter of a dental implant thread, the smaller the maximum equivalent stress, resulting in more effective stress distribution [28,29,30,31]. During masticatory movements, our teeth are subjected to combined loads, and torsional loading is one of the primary causes of dental implant failure [32,33]. Therefore, in this study, the maximum torque and weak points in dental implant structures to torsional loading were set as the QOI, and the structural stability of dental implants was investigated under COU.

The mechanical test to determine the torsional strength of dental implants showed that the rigid-type (short) model with an integrated abutment and screw was the worst case, with a yield strength of 0.61 Nm. The low structural strength of the rigid type may be attributed to the absence of a hexagonal shape when combined with the abutment and fixture.

Various loading and contact condition variables were applied for harmonizing CM&S with the mechanical performance test to present the ideal CM&S conditions. As for the finite element analysis program, Abaqus (ABAQUS, Dassault System, vélizy-villacoublay, France) was employed. This is one of the most widely used programs in various industries worldwide, considering its user-friendly accessibility. When testing the torsional strength of dental implants using CM&S, the most ideal conditions for harmonization were established. These conditions involved constraining the degree of freedom of the fixture’s external thread in all directions to resemble the mechanical test environment. Additionally, a rotational displacement was applied only to the widest diameter of the abutment. And small-sliding, surface-to-surface internal contact conditions were applied.

To create the same CM&S environment as the mechanical test environment, this study attempted to obtain ideal results by using a jig. However, when comparing the models with and without a jig, the models with a jig showed an error rate of up to 48% compared to the experimental values. In contrast, those without a jig showed an error rate under 5%. This means that the jig and the implant are perfectly combined during the mechanical test by applying pressure. However, in the CM&S environment, the abutment was not fully loaded, as the surfaces between the jig and the implant were different, thereby failing to achieve perfect contact. The load was fully applied to the abutment when there was no jig, resulting in similar results to the mechanical test. In addition, confirming the peak von Mises stress (PVMS) was critical. This confirmation indicated that the ideal CM&S environment was implemented, as the maximum PVMS appeared at the same location as the specimen fracture after the mechanical test.

### 4.2. Part 2: Total Knee Replacement (TKR)

In the same geometry, the coefficient of friction is the parameter that has the most significant impact on the maximum constraint in TKRs. This is generally predictable because, under the same vertical load, the greater the friction force, the greater the force required to move an object. However, the coefficient of friction between CoCr alloys and UHMWPE is known to range from 0 to 0.16 during walking, although it varies depending on the literature source [34]. It shows the applicability to the design of articular surfaces of femoral components and inserts because the force required to dislodge the artificial knee joint in the body can be predicted through analysis using the developed TKR M3DT.

The boundary conditions for *Y*-axis (medial–lateral) movement and *X*-axis (valgus–varus) rotation, which are the main movements in the knee joint, also affected TKR constraint. The rotational degree of freedom for the *X*-axis (valgus–varus) had a greater effect on TKR constraint than the effect of *Y*-axis (medial–lateral) movement. The difference between these boundary conditions seemed to affect the contact pressure and contact area between the femoral component and the insert. Although a difference was observed in the displacement where the maximum constraint occurred depending on each boundary condition, this phenomenon occurred because the maximum constraint was calculated in the section where the highest pressure was generated. However, these results may vary depending on the geometry and may help to confirm the designer’s intention.

The elastic modulus of the insert material (UHMWPE) was the parameter that had the least influence on the constraint results. This was because the constraint test was performed within the insert’s elastic zone. The peak von Mises stress (PVMS) applied to the insert in the constraint test was about 30 MPa, which had a minimal effect on the constraint. According to the literature, the reason is that the insert’s elastic modulus is about 3% of 900–1100 MPa [22,23]. This indicates that the risk of material properties in computer modeling of TKR constraint tests is relatively low.

This study has some limitations. First, it did not consider knee joint geometry. Variables such as the curved geometry between the femoral component and the insert, the insert height, and the size of the artificial knee joint should be studied further. Second, it conducted a quasi-static analysis without considering time. However, the results help to identify the influence of key parameters that should be considered in the computer modeling and simulation process as an M3DT.

## 5. Conclusions

Many parameters must be considered in computer modeling and simulation. Understanding how each parameter affects the results in advance increases the reliability of the simulation results and lowers the risk of the results. This study established the ideal loading and contact conditions for determining total knee replacement and dental implants. It also presented a reliable M3DT based on CM&S through harmonization. The findings of this study will help to apply reliable CM&S techniques to improve product performance, safety, and reliability throughout the entire life cycle of medical devices. This will contribute to the development of efficient and reliable product designs, establishing an effective M3DT.

## Figures and Tables

**Table 1:** Material properties of FE model.

Model	Material	Young’s Modulus (MPa)	Poisson’s Rate	Reference
Abutment	Ti-6Al-4V alloy	Nonlinear	0.35	[17]
Abutment Screw				
Fixture	Titanium grade 4	Nonlinear	0.34	[18]
Hydraulic Grip	Stainless steel	207,000	0.3	[19]
Collet	Spring steel	210,000	0.3	[20]

**Table 2:** Details of FE dental implant model.

Components	Element Type	Number of Node	Number of Element	Element Size (mm)
Abutment (rigid, long)	Tetrahedral (C3D4)	24,803	128,672	0.2
Abutment (rigid, short)		18,274	93,836	
Abutment (2-piece, long)		17,601	80,544	
Abutment (2-piece, short)		12,209	54,754	
Abutment (angled, 15°)		18,041	88,784	
Abutment (angled, 25°)		18,226	89,182	
Abutment screw		9822	48,395	
Fixture		20,379	95,225	
Hydraulic grip		62,428	332,308	0.5
Collet		39,194	191,212	

**Table 3:** Parameters of loading and boundary conditions.

Case	Abutment Loading Area	Sliding Contact		
		Area	Sliding Formulation	
			Finite Sliding	Small Sliding
			Node to Surface	Surface to Surface
1	All	Fixed	O	
2	All	Fixed		O
3	All			O
4	Part			O
5	Part		O	
6	Part			O

**Table 4:** Material properties of TKR FE model.

Model	Material	Young’s Modulus (MPa)	Poisson’s Rate
Femoral Component	Co-Cr-Mo Ally	21,000	0.33
Baseplate			
Jig			
Rail			
Insert	UHMWPE	900	0.42

**Table 5:** Parameters of boundary conditions.

Case	Boundary Condition		Elastic Modulus (MPa)	Friction Coefficient (μ)
	*X*-Axis (Medial–Lateral)	*Y*-Axis (Valgus–Varus)		
1	Fixed	Fixed	900	0~0.3 (0.05 units)
2	Fixed			0.05
3		Fixed		
4				
5				
6			855, 900, 945	0.15

**Table 6:** Mechanical test results of dental implant torsional strength.

Components	Yield Angle (°)	Yield Torque (Nm)
Rigid type (Long)	6.31 ± 0.25	0.81 ± 0.01
Rigid type (Short)	3.35 ± 0.60	0.61 ± 0.05
2-piece type (Long)	6.02 ± 0.07	0.77 ± 0.03
2-piece type (Short)	5.01 ± 0.09	0.92 ± 0.02
Angled type (15°)	5.02 ± 0.09	0.97 ± 0.02
Angled type (25°)	5.10 ± 0.26	0.95 ± 0.03

**Table 7:** Harmonization results according to loading and sliding contact condition variables.

Model	Results	Case					
		1	2	3	4	5	6
2-piece type (long)	FEA (Nm)	0.15	0.80	0.20	0.78	0.15	0.20
	Mechanical test (Nm)	0.77					
	Error rate (%)	−80.5	3.9	−74.0	1.3	−80.5	−74.0

**Table 8:** Comparison between experiment and FEA with jig: (a) comparison between experiment value and FEA without jig; (b) comparison between experiment and FEA with jig.

		**Model (a)**					
		**Rigid** **(Long)**	**Rigid** **(Short)**	**2-Piece** **(Long)**	**2-Piece** **(Short)**	**Angled** **(15°)**	**Angled** **(25°)**
Yield angle (°)	Experiment	6.07~6.70	2.54~4.19	5.98~6.14	4.87~5.11	4.89~5.61	4.92~5.17
	Average	6.31	3.35	6.02	5.01	5.10	5.02
	FEA	6	3	6	5	5	5
	Error Rate (%)	−4.91	−10.45	−0.33	−0.19	−1.96	−0.39
Yield Torque (Nm)	Experiment	0.78~0.82	0.53~0.66	0.75~0.81	0.91~0.95	0.90~0.98	0.94~0.98
	Average	0.80	0.61	0.77	0.92	0.95	0.97
	FEA	0.81	0.60	0.78	0.94	0.96	0.95
	Error Rate (%)	1.23	1.64	1.29	2.17	1.05	−2.06
		**Model (b)**					
		**Rigid** (**Long**)	**Rigid** (**Short**)	**2-Piece** (**Long**)	**2-Piece** (**Short**)	**Angled** (**Long**)	**Angled** (**Short**)
Yield angle (°)	Experiment	6.07~6.70	2.54~4.19	5.98~6.14	4.87~5.11	4.89~5.61	4.92~5.17
	Average	6.31	3.35	6.02	5.01	5.10	5.02
	FEA	6	3	6	5	5	5
	Error Rate (%)	−4.91	−10.45	−0.33	−0.19	−1.96	−0.39
Yield Torque (Nm)	Experiment	0.78~0.82	0.53~0.66	0.76~0.80	0.91~0.95	0.90~0.98	0.94~0.98
	Average	0.80	0.61	0.77	0.92	0.95	0.97
	FEA	0.47	0.75	0.40	0.64	0.61	0.76
	Error Rate (%)	−41.25	22.95	−48.05	−30.43	−35.79	−21.65

**Table 9:** Results of FE model.

Components	Element Type	Number of Nodes	Number of Elements	Element Size (mm)
Femoral Component + jig	Tetrahedral (C3D10)	60,137	40,182	4
Baseplate + jig		40,542	25,971	2.5
Insert		31,243	20,580	5
Rail		686	357	8

## Data Availability

The data presented in this study are available on request from the corresponding author.
